# Implementing Blockchains for Efficient Health Care: Systematic Review

**DOI:** 10.2196/12439

**Published:** 2019-02-12

**Authors:** Anuraag A Vazirani, Odhran O'Donoghue, David Brindley, Edward Meinert

**Affiliations:** 1 Healthcare Translation Research Group Department of Paediatrics University of Oxford Oxford United Kingdom; 2 Global Digital Health Unit Department of Primary Care and Public Health Imperial College London London United Kingdom

**Keywords:** blockchain, electronic health records, efficiency, interoperability, health, information science, computers

## Abstract

**Background:**

The decentralized nature of sensitive health information can bring about situations where timely information is unavailable, worsening health outcomes. Furthermore, as patient involvement in health care increases, there is a growing need for patients to access and control their data. Blockchain is a secure, decentralized online ledger that could be used to manage electronic health records (EHRs) efficiently, therefore with the potential to improve health outcomes by creating a conduit for interoperability.

**Objective:**

This study aimed to perform a systematic review to assess the feasibility of blockchain as a method of managing health care records efficiently.

**Methods:**

Reviewers identified studies via systematic searches of databases including PubMed, MEDLINE, Scopus, EMBASE, ProQuest, and Cochrane Library. Suitability for inclusion of each was assessed independently.

**Results:**

Of the 71 included studies, the majority discuss potential benefits and limitations without evaluation of their effectiveness, although some systems were tested on live data.

**Conclusions:**

Blockchain could create a mechanism to manage access to EHRs stored on the cloud. Using a blockchain can increase interoperability while maintaining privacy and security of data. It contains inherent integrity and conforms to strict legal regulations. Increased interoperability would be beneficial for health outcomes. Although this technology is currently unfamiliar to most, investments into creating a sufficiently user-friendly interface and educating users on how best to take advantage of it would lead to improved health outcomes.

**International Registered Report Identifier (IRRID):**

RR2-10.2196/10994

## Introduction

Medical records in Britain comprise of legacy paper records and numerous disconnected electronic systems. Despite the advancement of other health care fields such as oncology and neurology in their use of technology [1,2], there remains a lack of interoperability in health care systems, arising from the nonuniform record storage methods, that restricts doctors in their capacity to provide appropriate care [3]. Furthermore, the lack of (correct) information has been considered the primary cause of problems in health care, leading to medical errors and adverse events [4]. Not only does this include clinical errors, but administrative ones, such as the recent failure by the National Health Service (NHS) to invite almost 50,000 women for their cervical screening tests [5,6]. Patients must recount their history multiple times, which may be done incompletely. They too appreciate that interoperability would be beneficial in alleviating these adverse events [7,8]. The NHS planned to mend the situation by computerizing all records by 2018, however this target was delayed first to 2020 and again to 2023 [9]. In the United States, almost 90% of physicians already use a computerized system [10], although these are not all interoperable. Efficient health information technology (IT) systems are especially important at a time when the NHS faces shortages of more than 100,000 doctors and nurses [11,12], a total that is expected to increase with increasing demands on the service. Blockchain could solve the problem of interoperability by allowing doctors to gather information about a patient from multiple independent systems.

A blockchain is a decentralized online ledger (database), first implemented to store an ever-increasing record of all transactions using the cryptocurrency, Bitcoin [13]. It works by replacing trusted third-party signatories of a transaction (in a financial context, typically a “middle-man” payment provider such as Visa) with computational (cryptographic) proof to validate transactions. This validation is carried out by a network of users (“full nodes”) who collectively adhere to previously agreed rules, which are implemented by the software. This method saves both the cost of mediation, as a blockchain involves no mediator, and the cost associated with reversing transactions when disputes arise, as blockchain transactions are essentially irreversible. The transaction records are grouped into blocks, each of which is locked to the next with a cryptographic hash. Once recorded, data in any given block cannot be modified without altering all subsequent blocks (as each block’s hash depends on the last), nor without the agreement of a majority of the members of the network. As well as in financial services, distributed ledger technology has also been applied in the manufacturing industry to track goods within a supply chain [14,15], in governments for voting and public records [16,17], and in retail for unmediated goods trading and to allow more sophisticated loyalty packages [18,19].

The system is also flexible enough to allow the addition of arbitrary logic to process, validate, and access the data. This is implemented via components of business logic known as smart contracts, which reside on the blockchain and are synchronized across all nodes. A smart contract is a string of computer code that executes whenever certain conditions are met, ensuring security and authorized access [20]. The ability to create smart contracts makes blockchain suitable for health care, where strict regulations govern how sensitive data can be used [21,22]. Information exchange using smart contracts is transparent and conflict-free and eliminates the need for a middleman, as the blockchain executes the data sharing based on the preagreed conditions of the contract [23,24]**.**

Ownership and privacy of data are important issues that blockchain could solve. It is currently debated whether the health care provider or the patient owns health care data relating to a patient (although patients have a definite right to access the data [25]). In addition to ownership issues, with the introduction of the General Data Protection Regulation (GDPR) in the European Union, it is important for patients to know how their personal information is being handled [21,26]. Smart contracts implemented by a blockchain would simplify the consent process for data access by doctors. The current consent process is not standardized or personalized, which makes it difficult for a patient to express clearly via an access control policy, which may, for example, involve allowing selected access to particular specialists.

Another concern with medical records is the cost currently associated with transferring records between locations [27]. Repeated imaging studies carried out because of unavailability of prior results can be dangerous in terms of delayed treatment as well as financially costly. Sending data via email is considered a security risk [28,29], and there is clear inefficiency inherent in transcribing a digital asset onto optical media which is commonly read only once at the receiving site [22,30]. A system integrating patient consent as well as access to authorized individuals would save on these costs.

Medical information is no longer limited to written reports, imaging studies, and blood tests. Genomic data and that collected by wearable devices, such as bracelets and watches embedded with sensors, are increasingly accumulated. If exploited effectively, the availability of these new forms of data may lead to improved treatment options and outcomes and may also be examined by health insurance companies offering discounts for “healthy” behavior. Further benefits arise in the realm of artificial intelligence. When given the appropriate data, this can infer trends from the data that are then used to generate population-level insight, and so achieve population health as a whole. These new data formats, however, will require careful integration to allow appropriate analysis while maintaining patient privacy and security against hackers.

Although digitization of health records has been in place in the general practitioner (GP) sector for over 30 years (albeit lacking essential data sharing and exchanging capabilities), secondary care has not yet successfully achieved this de facto standard. Distributed ledger technology, initiated and exemplified by the bitcoin blockchain, is having a growing impact on IT environments in which conformation to legislative regulations and maintenance of public trust are increasingly paramount [31], and it may be used in realizing NHS Digital’s target. The aim of this review was to summarize the evidence relating to the implementation of blockchains to manage electronic health records (EHRs), and to discuss whether this would improve efficiency of record management.

## Methods

The review protocol [32] was used with the following modifications: (1) The research question was narrowed to focus on efficiency, as the issues of privacy and scalability would broaden the review excessively, (2) A total of 5 additional search databases were included to account for the potential lack of published research on the topic, and (3) The population was extended to anyone whose health data are stored in a blockchain.

### Research Question and Definitions

RQ: What strategies have been proposed or trialed to implement a blockchain or blockchains for the management of electronic medical records, and how do they improve efficiency compared to currently employed medical record management methods?

1. *Medical record*: [any] record consisting of information about the physical or mental health or condition of an identifiable individual made by or on behalf of a health professional in connection with the care of that individual [33].
2. *Efficiency*: Either improved interoperability or cost-effectiveness, or improved health outcomes as a result of these.
3. *Current methods*: These may consist of traditional paper-based methods or more advanced technology adopted to provide more coordinated health care.
4. *Interoperability*: Automatic and seamless exchange of health information across health information systems [34]. (This corresponds to syntactic interoperability.)

### Ethics and Dissemination

As data collection was executed via published literature, ethical approval was not be required for this review.

### Search Strategy and Study Selection

We searched PubMed, Scopus, EMBASE, MEDLINE, ProQuest, CINAHL, AMED, Global Health, Books@Ovid, and Cochrane Library for all relevant literature. We also searched for other systematic reviews on the topic on the PROSPERO [35] database. We used the MeSH database [34] to derive keywords and search term combinations (Multimedia Appendix 1). As blockchains applied to the health care sector remain a novel approach, we did not place restrictions on the study type, dates of publication, or geographic locations. However, only studies in English were included.

Results of the search strings (Multimedia Appendix 2) were imported into EndNote X8.0.1 (Clarivate Analytics), which was used to remove duplicate articles. Remaining duplicates were deleted manually. We used an iterative approach of title, abstract, and full text searches (Multimedia Appendix 3) and exported the results into Microsoft Excel. Titles and abstracts were then independently screened by 2 reviewers, based on the following inclusion criteria:

1. Articles must discuss the use of blockchain to manage medical records in some manner, and
2. Articles must describe the benefits and/or disadvantages of using this technology (and compare this to currently used methods of managing medical data).

Where the second point may not be determined from the abstract alone, the study should be taken to full text screening. Studies may not identify a comparator, and these may be included provided the remaining inclusion criteria are met.

**Figure 1:**
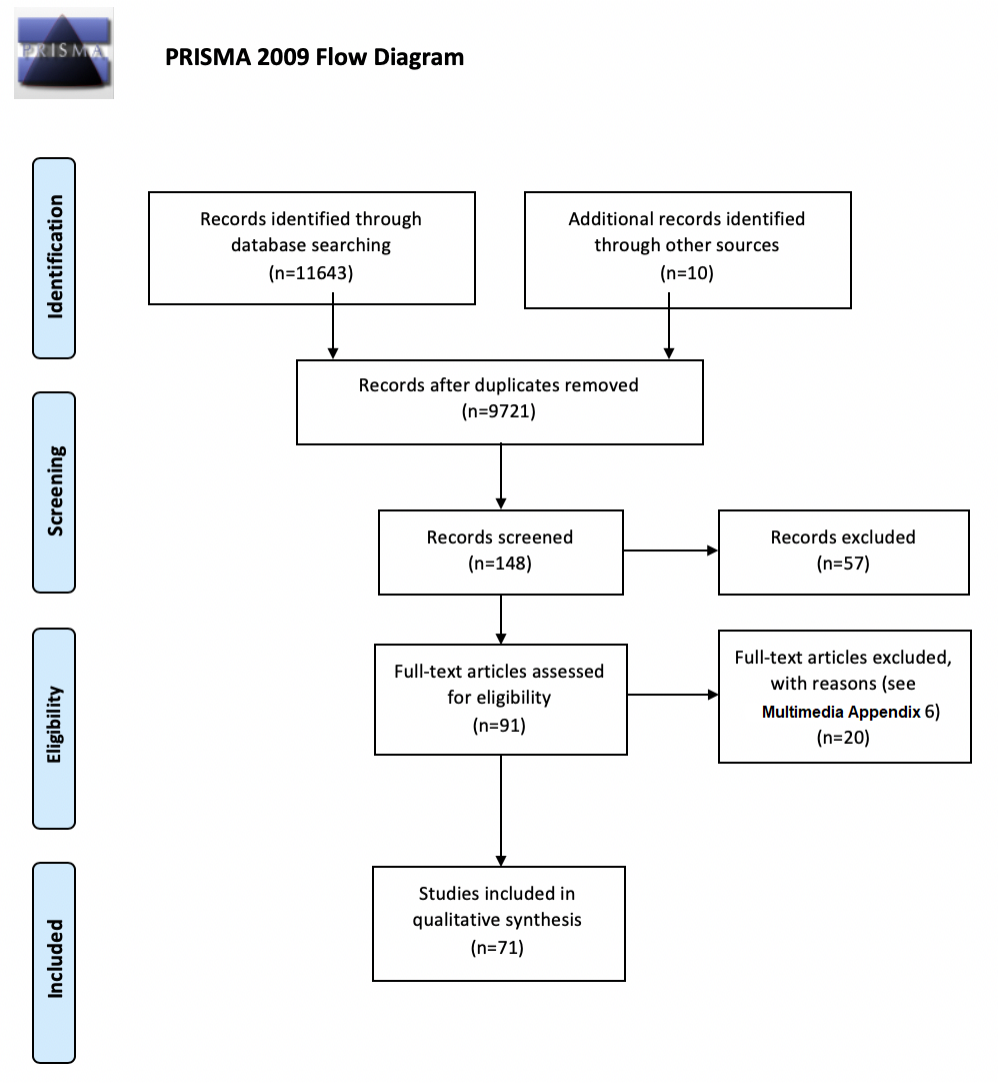
Preferred Reporting Items for Systematic Reviews and Meta-Analyses flow diagram.

A total of 2 reviewers (AV and OO) resolved discrepancies through discussion (Multimedia Appendix 4), and no adjudication from a third reviewer was required. The full texts of the remaining articles were subsequently assessed for their eligibility, based on the same eligibility criteria. This selection process is demonstrated using the Preferred Reporting Items for Systematic Reviews and Meta-Analyses flow diagram (Figure 1).

### Data Extraction

A template was designed to collect information required to address the research question. Basic metadata were collected automatically by EndNote, and the remaining data items (Multimedia Appendix 5) were gathered after reading the papers in full.

### Outcomes

The primary outcome measures were interoperability and cost-effectiveness (our definition of efficiency). The secondary outcome measure was improved health outcomes, although it was noted that it might be difficult to determine a quantitative measure of this with respect to blockchains.

### Strength of Evidence and Data Synthesis

Studies of interventions involving randomized and nonrandomized methods were assessed for risk of bias using the Cochrane Collaboration Risk of Bias Tool and the Risk of Bias in Nonrandomized Studies - of Interventions tools, respectively.

The extracted data were subsequently summarized qualitatively. No meta-analysis was performed, because application of blockchains in health care remains a novel method and articles with sufficient numerical data were not found. In addition, the heterogeneity of studies prevented a meta-analysis.

## Results

### Description and Characteristics of Included Studies

After the initial literature search, removal of duplicates, eligibility (Multimedia Appendix 6), and full-text screening, 61 articles were included in the study. An additional 10 articles were added via snowballing (review of the references from the included articles) of the full texts screened (Table 1).

Few studies described the implementation of a blockchain system to real-world medical data, highlighting the novelty of this technique. Of these, 1 [36] used smart contracts to manage access to medical data that were stored on the cloud, whereas the others stored medical data directly on the blockchain. Of the largest group of articles which proposed a framework (without testing it on live data), the majority advocated cloud-based data storage with blockchain-based access control. In addition to the primary outcome of interoperability, issues considered in these articles included those of privacy and data security, scalability, and administrative affairs. There are also a number of companies currently implementing blockchains in health care, which were referred to throughout the literature, and many of which have not published any academic literature. These are collated in Multimedia Appendix 7.

**Table 1:** Summary themes of included articles.

Article type	Number of articles
Implementation of EHR^a^ blockchain system (tested on real data)	4 [36-39]
Proposal of EHR blockchain framework (not tested on real data)	18 [21-23,31,40-52]
Discussion of benefits and drawbacks of the use of blockchain for EHRs	10 [8,24,28,53-59]
Description of company in the field	20 (Multimedia Appendix 7)
Newspaper, magazine, and columns (opinion articles)	19 in searched databases

^a^EHR: electronic health record.

The majority of the information comparing blockchain’s potential versus current methods of managing records was found in opinion articles, which were set more broadly in the context of developments in health care technology; many described the disarray of current health record management. Some used the successes of blockchain in other fields than health care and finance to demonstrate its versatility.

### Outcome Measures

Interoperability, one of our primary outcome measures, was seen as feasible using a blockchain approach to store an index of records and to manage access to cloud-based records [36,51]. This approach saves the administrative costs involved in transporting records, as well as the medical costs associated with waiting for their arrival. Logistical difficulties and costs may arise in collating legacy data, although we expect that these would be accounted for in savings from improved health outcomes in the long term.

## Discussion

### Summary of Evidence

A blockchain can allow improved interoperability as data across multiple systems can be exchanged and accessed simultaneously and immediately. The interfacing of different systems would also save costs, as no expensive data mediators or escrow services are required to broker trust [8,45]. Costs are also saved in the process of data transfer, which becomes immediate [27]. This is especially useful in the management of patients suffering from chronic diseases, facilitating the delivery of care by connecting both patients and providers with medical data. These factors, therefore, reduce administrative delays, as does the use of smart contracts to execute patients’ consent preferences immediately [23,24]. An off blockchain data repository (“data lake”) is scalable and can store a variety of data types, as well as being a tool for research. This is in contrast to current health care data systems, which are not synchronized to allow intersystem communication or equipped to manage emerging data formats [9]. Blockchain is interactive and supports high throughput data analysis and machine learning, while being encrypted and digitally signed to ensure data privacy and authenticity [51]. These are particularly important factors in a field that is both dominated by evidence-based research but bound by strict data regulations [21]. The disjointed systems currently in use do not lend themselves toward such modern research techniques, nor are they stored in a secure or immutable manner. The collaboration between patients, doctors, and researchers arising from a blockchain-based system allows for a greater degree of exchange and comparison, leading to specific and personalized care pathways [31,45]. The Office of the National Coordinator for Health Information Technology (ONC) has recently described several features critical to the development of an interoperable health system, which are addressed by blockchain [60], such as establishing a directory of resource locations that can be easily referenced to locate information and creating an economic environment in which interoperability is a sound business decision[22].

### Interoperability

Health data are dynamic and expansive, and seamless exchange of health data across health information systems would be advantageous. As it would not be practical in terms of speed, storage capacity, or sustainability to replicate all health records on every computer in the blockchain network [54], we instead advocate blockchain as a method to manage access control (and for smart contract management) by systematically storing a catalogue of all users’ health records and related metadata. Each time data are added to the EHR by a doctor or patient (from a mobile app or wearable sensor), a metadata-containing pointer to this is added to the blockchain, while the data are stored securely on the cloud [51]. A full index of a particular patient’s records is stored in a single location along with related metadata, regardless of the whereabouts of the medical data. The blockchain, with this secured index of records, then directs authorized individuals to the cloud-based data, thus allowing the immediate exchange of information between approved professionals, while also keeping an immutable ledger of those readers. The fact that blockchain is based on open-source software also has potential benefits, as health trusts can use the open application programming interface to integrate data as they wish, giving them timely access to accurate information in a format useable by them. Striving for interoperability is also a key feature of the Health Information Technology for Economic and Clinical Health Act, which has meant that since 2011; American health care providers have been given financial incentives to demonstrate meaningful use of EHRs [61].

### Health

Fast access to a comprehensive set of patient data would allow doctors to treat patients without the need to wait for the arrival of previous results. The availability not only of prompt but more frequent data would allow physicians to create specialized treatment plans on the basis of outcomes and treatment efficacy. Daily health data would also engage a patient more in their own health care, and improve patient compliance [51], a historic challenge in the field [62]. The capability of personalized medicine would, therefore, be improved with this interoperability, as a single access point for all real-time health data is created for each patient. Data gathered from wearable sensors and mobile apps would contribute information on the risks and benefits of treatments, and on patient-reported outcome measures.

### Integrity

The immutability of a blockchain that stems from linking the hashes of subsequent blocks, carries with it inherent integrity as blocks cannot be rewritten without collaboration of a majority of nodes. This is key to maintaining a true record of patient-provider interactions as well as data originating from devices, both of which could influence not only medical decisions but also those involving insurance. This property was exemplified by RadBit at the 2017 Yale Healthcare Hackathon [46], a system which allows patients to keep possession of their medical images, along with an immutable chain of custody. Temporary keys (“tokens”) can be created by users of the blockchain and passed onto those such as health care providers and insurance companies, providing them temporary access. The token is independent of the data, containing only authorization commands, and is verified and validated (by recording them on the chain) before the required reports are dispatched. Integrity may also be maintained by the use of external auditors, who may verify the accuracy of the system in real time and retrospectively [21]. Potential ways to improve the integrity are to use blind signatures, which reinforce protection from tampering as well as confirming the sender’s and viewer’s identities [53], or to use signatures from multiple authorities [42].

### The Office of the National Coordinator for Health Information Technology Blockchain Challenge

In 2016, the ONC organized the “Use of Blockchain in Health IT and Health-Related Research” challenge, seeking ideas to address the difficulties of managing health records [20,63,64]. Winning papers described innovative ways to securely empower patients through interoperability [57,65]. MedRec, one of the winning entries, is now being implemented in Boston. This proposal from Massachusetts Institute of Technology’s media lab involves associating a medical record with viewing permissions and data retrieval instructions for execution on external databases, thus using the blockchain to record patient-provider interactions via smart contracts. Once a doctor creates a record, it is verified, and its viewing permissions are authorized by the patient. The party receiving new information receives an automated notification [3] and a hashed pointer to the new medical record, and its permissions are stored on the chain. This system allows patients to be empowered through access and control of their data, options which have until recently not been available outside a trial setting [57], and which we hope will soon become a standard of care. So far, their system has been successful with medications, blood tests, vaccination histories, and other therapeutic interventions [66].

### Large-Scale Implementation

Blockchain has recently been adopted on a large scale by the Estonian government, in collaboration with Guardtime, where it now secures millions of records. Other companies involved in introducing blockchain to everyday health care include Medicalchain, which allows users to sign up and use the interface to interact with their GP, and Patientory that connects doctors, health providers, and consumers, and others listed in Multimedia Appendix 7.

### Challenges and Limitations

#### Data Ownership and Privacy

Achieving interoperability depends on patients taking control of their data and deciding on how it is to be used, features integral to blockchain. Although shifting data ownership from the government and companies to patients would require extensive reengineering of legacy systems to introduce a blockchain, it would incentivize patients to become active agents in their own care by contributing data to get the best possible treatment [8,67,68]. The blockchain would also give patients the sole power to authorize data access to various providers at their discretion [41], eliminating delays associated with the current bureaucracy [52,60,69], and ensuring patient privacy [21]. These benefits bestowed by blockchain stand to empower patients with control over their data, a new expectation in a time when mutualistic and consumerist patient-doctor relationships are becoming the norm. Patients could also selectively share data with researchers using blockchain, either for the greater scientific good or to enable studies on their unique condition [70]. The system would guarantee their consent, a factor key to establishing autonomy and patient-centered care. A recent example of patient autonomy over health data is 23andMe, 80% of whose users chose to make their genomic data available to researchers. This demonstrates that patients will be happy to share data for research should they stand to benefit. Enabling direct patient involvement in controlling the use of their records in this open and secure manner enabled by blockchain will enhance the uptake of such platforms and potentially lead to improved health outcomes [31]. Sharing data may be accomplished with a trusted system, but as an additional incentive, there is the idea of “rewarding” patients for healthy behavior, such as with lower insurance premiums [21,56]. This and other similar ideas will need to be piloted to determine how effectively they will overcome this challenge.

#### Legal

Under GDPR (Article 17), the Organization of Economic Cooperation and Development privacy guideline, the Health Insurance Portability and Accountability Act (HIPAA) Privacy Rule, and others [58], individuals may request for their data to be erased. This is possible when the records themselves are not stored on the blockchain. However, a record of the data’s previous existence may still be maintained within the chain, even if the data were to be deleted. The legal question arising from this relates to whether metadata of personal data classifies as personal data [54]. Regardless, the fact that data in the system would in any case be controlled by the patient is a definite sign of progress. A potential limitation here arises with respect to the use of a private or consortium-led blockchain, as these data privacy concerns would need to be addressed to make the services compliant and vendor neutral. This may be achieved by having a governing authority enforce open standards. Although these sorts of regulatory constraints are necessary to ensure such appropriate use of information, they may slow development in the field. HIPAA, for example, requires that an institutional review board approve the use of data [21]. That any such delay would be substantial is however unlikely, and so compliance with these regulations is paramount to the success of blockchain.

#### Security

Sensitive data must be kept safe from eavesdroppers and intruders [41,71]. Breaches have a negative impact on the public perception of the health care field and threaten to hinder future research through more stringent regulatory restrictions [72,73]. The WannaCry attack of May 2017 infected many thousands of computers worldwide, including those of the NHS [64,74,75]. One earlier attack in Los Angeles targeted EHRs in particular, demanding thousands of dollars in ransom [76]. A blockchain is more secure than legacy methods, which would issue patients with credentials [23]. It achieves this property by the use of public-key cryptography (as opposed to symmetric-key cryptography, the method typically used for encryption). This involves generating a public and private key for each user using a one-way encryption function, known as a hash. There is no way for anyone but the recipient to see information sent over the blockchain, as it is secured by their private key. The only security flaw that may arise with a blockchain is unrelated to encryption and comes about if a public blockchain is used: hackers could collude in a “51% attack,” resulting in the rewriting of the chain structure [77]. Thus, to realize the advantages of a decentralized system, patients must have some trust that at least 50% of mining nodes would not want to violate the immutability of the blockchain. The public blockchain also leads to the possibility of patient identification, which would need to be avoided by pseudonymizing data to protect patients’ identities [22,56]. If a private or consortium blockchain was to be used, however, mining nodes would be limited to hospitals and other trusted health providers, eliminating these security flaws.

#### Other Concerns

Although the major concerns with blockchain are those of security, privacy, and legal restrictions, for which various workarounds have been developed, there remain some further challenges to consider. First, consolidating data from legacy systems will involve removing data that are duplicated in different parts of the system [8] and converting outdated file formats. This introduces an implementation cost, in excess of the basic cost to introduce a blank system [78], which a government may not be willing to spend [79]. Second, as with any system, it is necessary for users to input good quality information: the trustworthiness arising from blockchain’s immutability and decentralization concedes to the input of low-quality (incorrect) information [8,40]. Finally, the issue of currency, used in blockchain to incentivize users to mine blocks in the new network. An initial coin offering [45,57,80] could initiate this process by valuing the new token as funds are raised. However, extremes of price could deter miners, and so mining may need to be restricted to health care providers to avoid this. Another view is to remove all currency, as data are owned by the patient and are not inherently an exchangeable currency [23]. On the basis of this, we may assume that providers already have an incentive to secure patients’ medical information, and so there would be no need to incentivize mining beyond the simple use of the system. Finally, the reliance of a blockchain on essentially arbitrary computation could be seen to introduce administrative inefficiency [31]. Transactions are therefore energy-intensive, as each must be computationally verified and validated by the whole network [45]. However, such a mechanism is still beneficial, as rather than providing economic value it demonstrates proof-of-participation, which would be required for ongoing use of the system.

### Conclusions

The storage and sharing of medical data (developing interoperability) are vital for improved health outcomes. Respecting privacy of sensitive information while doing this remains a big challenge in health care. The literature shows that with the appropriate regulatory guidelines and use standards, blockchain can act as a vehicle to manage consented access to EHRs. This will increase interoperability without compromising security, while also protecting patient privacy. These issues would most effectively be tackled by the use of a private or consortium-led blockchain; however, this would need to be regulated to ensure appropriate use of data. The improved interoperability and reduced long-term administrative costs would lead to improved health outcomes.

Blockchain represents a new form of technology in which the current literature is lacking in this application context, and no usage feedback or statistical comparisons with traditional systems exist. There are costs associated with transferring to a new system, and in educating health professionals and patients on how best to take advantage of it for improved health. Blockchain involves concepts unfamiliar to the vast majority of the population, such as cryptographic signature and key management. Investments into new systems would, however, be outweighed through returns. In the primary stages of implementation, the practical usefulness of the proposed system will likely depend on the end-user experience—the complexities underlying the blockchain will need to be hidden behind a sufficiently user-friendly interface such as an online or mobile app to be adopted successfully. Short-term trials will outline the most effective ways to implement such a user-friendly experience, which may be expanded thereafter.

## Appendix

Multimedia Appendix 1 Search terms and keywords.

Multimedia Appendix 2 Search strings.

Multimedia Appendix 3 Electronic screening.

Multimedia Appendix 4 Title and abstract screening.

Multimedia Appendix 5 Data extraction items.

Multimedia Appendix 6 Eligibility stage search exclusions.

Multimedia Appendix 7 Blockchain and company system names.

## Abbreviations

EHR: electronic health record
GDPR: General Data Protection Regulation
GP: general practitioner
HIPAA: Health Insurance Portability and Accountability Act
IT: information technology
NHS: National Health Service
ONC: Office of the National Coordinator for Health Information Technology

## References

[1] Sample I The Guardian.

[2] Ding Y, Sohn JH, Kawczynski MG, Trivedi H, Harnish R, Jenkins NW, Lituiev D, Copeland TP, Aboian MS, Aparici CM, Behr SC, Flavell RR, Huang S, Zalocusky KA, Nardo L, Seo Y, Hawkins RA, Pampaloni MH, Hadley D, Franc BL (2018). A deep learning model to predict a diagnosis of alzheimer disease by using F-FDG PET of the brain. Radiology.

[3] Brock J (2017). Healthcare Innovation.

[4] (2015). CRICO.

[5] Capita.

[6] Iacobucci G (2018). Cervical screening: GP leaders slam Capita over failure to send up to 48 500 letters. Br Med J.

[7] PwC UK.

[8] Engelhardt MA (2017). Hitching healthcare to the chain: an introduction to blockchain technology in the healthcare sector. TIM Rev.

[9] Department of Health & Social Care.

[10] Jamoom E, Yang N (2016). Centers for Disease Control and Prevention.

[11] The Nuffield Trust.

[12] Iacobucci G (2018). NHS staff shortages could reach 250 000 by 2030 without urgent action, experts warn. Br Med J.

[13] Nakamoto S (2008). Bitcoin.

[14] Skuchain.

[15] Wayback Machine.

[16] Aki J Bitcoin Magazine.

[17] Ruubel M Guardtime.

[18] OpenBazaar.

[19] Loyyal.

[20] Wright G, Wright A, Landman A (2017). NEJM Catalyst.

[21] Mamoshina P, Ojomoko L, Yanovich Y, Ostrovski A, Botezatu A, Prikhodko P, Izumchenko E, Aliper A, Romantsov K, Zhebrak A, Ogu IO, Zhavoronkov A (2018). Converging blockchain and next-generation artificial intelligence technologies to decentralize and accelerate biomedical research and healthcare. Oncotarget.

[22] Patel V (2018). A framework for secure and decentralized sharing of medical imaging data via blockchain consensus. Health Informatics J.

[23] Dagher GG, Mohler J, Milojkovic M, Marella PB (2018). Ancile: privacy-preserving framework for access control and interoperability of electronic health records using blockchain technology. Sustain Cities Soc.

[24] Alhadhrami Z, Alghfeli S, Alghfeli M, Abedlla JA, Shuaib K (2017). Introducing Blockchains for Healthcare.

[25] (2018). Nottinghamshire Local Medical Committee Ltd.

[26] Health Information Privacy.

[27] Patel V, Barker W, Siminerio E Office of the National Coordinator for Health Information Technology.

[28] Dubovitskaya A, Xu Z, Ryu S, Schumacher M, Wang F (2017). Secure and trustable electronic medical records sharing using blockchain. AMIA Annu Symp Proc.

[29] Health Information Privacy.

[30] Erickson BJ (2011). Experience with importation of electronic images into the medical record from physical media. J Digit Imaging.

[31] Cunningham J, Ainsworth J (2017). Enabling patient control of personal electronic health records through distributed ledger technology. Stud Health Technol Inform.

[32] Meinert E, Alturkistani A, Foley Kimberley A, Osama Tasnime, Car J, Majeed A, Van Velthoven Michelle, Wells G, Brindley David (2019). Blockchain Implementation in Health Care: Protocol for a Systematic Review. JMIR Res Protoc.

[33] National Health Service.

[34] MeSH Browser.

[35] PROSPERO.

[36] Azaria A, Ekblaw A, Vieira T, Lippman A (2016). MedRec: Using Blockchain for Medical Data Access and Permission Management.

[37] Ichikawa D, Kashiyama M, Ueno T (2017). Tamper-resistant mobile health using blockchain technology. JMIR Mhealth Uhealth.

[38] Lee SH, Yang CS (2018). Fingernail analysis management system using microscopy sensor and blockchain technology. Int J Distrib Sens Netw.

[39] e-Estonia.

[40] Dubovitskaya A, Xu Z, Ryu S, Schumacher M, Wang F (2017). How Blockchain Could Empower eHealth: An Application for Radiation Oncology.

[41] Omar A, Rahman M, Basu A, Kiyomoto S (2017). MediBchain: A Blockchain Based Privacy Preserving Platform for Healthcare Data.

[42] Guo R, Shi H, Zhao Q, Zheng D (2018). Secure attribute-based signature scheme with multiple authorities for blockchain in electronic health records systems. IEEE Access.

[43] Liang X, Shetty S, Zhao J, Bowden D, Li D ResearchGate.

[44] Liang X, Zhao J, Shetty S, Liu J, Li D (2017). Integrating Blockchain for Data Sharing and Collaboration in Mobile Healthcare Applications.

[45] Mannaro K, Baralla G, Pinna A, Ibba S (2018). A blockchain approach applied to a teledermatology platform in the Sardinian region (Italy). Information.

[46] Nichol PB (2017). CIO.

[47] Raju S, Rajesh V, Deogun JS (2017). The Case for a Data Bank: an Institution to Govern Healthcare and Education. Proceedings of the 10th International Conference on Theory and Practice of Electronic Governance.

[48] Siddiqi M, All S, Sivaraman V (2017). Secure Lightweight Context-driven Data Logging for Bodyworn Sensing Devices.

[49] Zhang J, Xue N, Huang X (2016). A secure system for pervasive social network-based healthcare. IEEE Access.

[50] Zhao Z, Zhang Y, Peng Y, Xu Ruzhi (2017). Lightweight backup and efficient recovery scheme for health blockchain keys.

[51] Linn LA, Koo MB Office of the National Coordinator for Health Information Technology.

[52] Yue X, Wang H, Jin D, Li M, Jiang W (2016). Healthcare data gateways: found healthcare intelligence on blockchain with novel privacy risk control. J Med Syst.

[53] Liu P (2016). Medical record system using blockchain, big data tokenization.

[54] Esposito C, de Santis A, Tortora G, Chang H, Choo KR (2018). Blockchain: a panacea for healthcare cloud-based data security and privacy?. IEEE Cloud Comput.

[55] Mertz L (2018). (Block) chain reaction: a blockchain revolution sweeps into health care, offering the possibility for a much-needed data solution. IEEE Pulse.

[56] Mertz L (2018). Hospital CIO explains blockchain potential: an interview with Beth Israel Deaconess Medical Center's John Halamka. IEEE Pulse.

[57] Pauwels E, Grevatt N (2017). Wilson Center.

[58] Weiss M, Botha A, Herselman M, Loots G (2017). Blockchain as an enabler for public mHealth solutions in South Africa.

[59] Wong MC, Yee K, Nøhr C (2018). Socio-technical considerations for the use of blockchain technology in healthcare. Stud Health Technol Inform.

[60] Office of the National Coordinator for Health Information Technology.

[61] Office of the National Coordinator for Health Information Technology.

[62] Scambler G (2008). Sociology as Applied to Medicine Sixth Edition.

[63] Office of the National Coordinator for Health Information Technology.

[64] Slabodkin G (2017). Blockchain remains a work in progress for use in healthcare. Health Data Manage.

[65] ID2020 Alliance.

[66] Journal of Ahima.

[67] Kitson A, Marshall A, Bassett K, Zeitz K (2013). What are the core elements of patient-centred care? A narrative review and synthesis of the literature from health policy, medicine and nursing. J Adv Nurs.

[68] Stewart M (2001). Towards a global definition of patient centred care. Br Med J.

[69] Potts J (2016). The big cure. IPA Review.

[70] Prajna S (2017). CommsMEA.

[71] Health Information Privacy.

[72] Patil HK, Seshadri R (2014). Big Data Security and Privacy Issues in Healthcare.

[73] Padmanabhan P CIO.

[74] Fox News.

[75] Recode.

[76] Conn J Modern Healthcare.

[77] Wood G Gavin Wood.

[78] Moss J, Smith C, Davies J (2017). Healthcare Dive.

[79] Padmanabhan P (2017). CIO.

[80] Damiani J (2017). Forbes.

