# Supplementary material for: Implementing Blockchains for Efficient Health Care: Systematic Review
Source: J Med Internet Res. 2019 Feb 12;21(2):e12439. doi: 10.2196/12439 (PMC6390185; doi:10.2196/12439)
Supplement: Multimedia Appendix 1 [file jmir_v21i2e12439_app1.docx]

Multimedia Appendix 1

| **Intervention/Outcome term** | **MeSH Heading** | **Similar search terms** |
| --- | --- | --- |
| Blockchain | N/A | Blockchain* |
| Efficiency | Efficiency | Organisation, organization, productivity |
| Health outcomes | Health | Normality |
| Cost effectiveness | Cost-benefit analysis | Cost-benefit data, cost-utility analysis, economic evaluation, marginal analysis, cost benefit, cost-effectiveness analysis |
| Electronic health records | Electronic health records | Computerised health records, computerized health records, computerised medical records, computerized medical records, electronic medical records, electronic healthcare records |
