# Supplementary material for: Implementing Blockchains for Efficient Health Care: Systematic Review
Source: J Med Internet Res. 2019 Feb 12;21(2):e12439. doi: 10.2196/12439 (PMC6390185; doi:10.2196/12439)
Supplement: Multimedia Appendix 2 [file jmir_v21i2e12439_app2.docx]

Multimedia Appendix 2

| **Database(s)** | **Exact search string** | **Number of results** |
| --- | --- | --- |
| Pubmed | "blockchain*"[All Fields] AND ("efficien*"[All Fields] OR "cost-benefit analysis"[All Fields] OR "EMR"[All Fields] OR "EHR"[All Fields] OR (("electronic"[All Fields] OR "computer*"[All Fields]) AND "record*"[All Fields])) | 6 |
| Scopus | ALL ( "blockchain*" AND ( "efficien*" OR "organis*" OR "organiz*" OR "cost*" OR "EMR" OR "EHR" OR ( ( "electronic" OR "computer*" ) AND "record*" ) OR "health*" OR "medic*" ) ) | 1365 |
| CINAHL | TX "blockchain*" AND ( "efficien*" OR "organis*" OR "organiz*" OR "cost*" OR "EMR" OR "EHR" OR ( ( "electronic" OR "computer*" ) AND ("record*" OR "data") ) OR "health*" OR "medic*" ) | 34 |
| Books@Ovid, AMED, Embase, Global Health, Medline (accessed together via Ovid) | "blockchain*" AND ( "efficien*" OR "organis*" OR "organiz*" OR "cost-benefit analysis" OR "EMR" OR "EHR" OR ( ( "electronic" OR "computer*" ) AND ("record*" OR "data") ) OR "health*" OR "medic*" ) {Including Limited Related Terms} | 5408 |
| Proquest | "blockchain*" AND ("health*" or "medic*") AND ("efficien*" OR "organis*" OR "organiz*" OR "cost*" OR "EMR" OR "EHR" OR (("electronic" OR "computer*") AND ("record*" OR "data") )) | 5483 |
| Cochrane Library (full text) | “blockchain*” | 0 |
|  | ("efficien*" OR "organis*" OR "organiz*" OR "cost*") AND ("EMR" OR "EHR" OR (("electronic" OR "computer*") AND ("record*" OR "data") )) | 14273 (excluded - see below) |

The Cochrane Library was searched for the term “blockchain”, but this returned no results. A search for the second string listed in the table returned 14273 results, including 7671 Cochrane Reviews, 1669 Other reviews, 3335 Trials, 122 Method studies, 115 Technology assessments, 1306 Economic evaluations, and 55 Cochrane groups. These were imported and screened using Endnote, with none containing the term “blockchain” in their full text, and so all were discarded.

A search for the term “blockchain” on PROSPERO returned only the protocol cited for the current systematic review.
