# Supplementary material for: Implementing Blockchains for Efficient Health Care: Systematic Review
Source: J Med Internet Res. 2019 Feb 12;21(2):e12439. doi: 10.2196/12439 (PMC6390185; doi:10.2196/12439)
Supplement: Multimedia Appendix 3 [file jmir_v21i2e12439_app3.docx]

Multimedia Appendix 3

| Term | Field | Criteria | Articles remaining | Notes |
| --- | --- | --- | --- | --- |
| “blockchain*” | Full text | Articles without blockchain in the entire text are unlikely to be relevant | 1546 | 8165 excluded |
| “health*” OR “med*” | Full text | Articles need to relate to blockchain specifically in healthcare | 688 | (317 health, 574 med), 858 excluded |
| “record*” OR “data” | Full text | Articles must relate to the use of blockchain specifically for EHR | 448 | (336 record, 320 data), 240 excluded |
| “blockchain*” | Abstract | Articles without blockchain in the abstract are unlikely to be relevant | 370 | 78 excluded |
| “health*” OR “medic*” | Abstract | Articles without some direct health or medical link in the abstract are likely to be focussed on other blockchain applications | 191 | (144 health, 96 medic), 179 excluded |
| REMOVE “financ*” | Abstract | Likely to be too related to cryptocurrency | 149 | 42 excluded (many of which would also have contained “currenc*”, see following screen) |
| REMOVE “currenc*” | Abstract |  | 138 | 9 excluded |
