# Supplementary material for: Implementing Blockchains for Efficient Health Care: Systematic Review
Source: J Med Internet Res. 2019 Feb 12;21(2):e12439. doi: 10.2196/12439 (PMC6390185; doi:10.2196/12439)
Supplement: Multimedia Appendix 4 [file jmir_v21i2e12439_app4.docx]

Multimedia Appendix 4

The following outlines the full text screening process carried out by two reviewers independently:

| Agreements - Yes | 69 |
| --- | --- |
| Agreements - No | 53 |
| Disagreements - AV Yes/OO No | 11 |
| Disagreements AV No/OO Yes | 5 |
