# Supplementary material for: Implementing Blockchains for Efficient Health Care: Systematic Review
Source: J Med Internet Res. 2019 Feb 12;21(2):e12439. doi: 10.2196/12439 (PMC6390185; doi:10.2196/12439)
Supplement: Multimedia Appendix 5 [file jmir_v21i2e12439_app5.docx]

Multimedia Appendix 5

| # | Data Item | Description |
| --- | --- | --- |
| 1 | Research question | What was the author’s research question(s)? |
| 2 | Sources | What data sources did they draw upon? |
| 3 | Analysis | What method of analysis did they use? |
| 4 | Results | What were the main findings? |
| 5 | Conclusion | What is their conclusion |
