# Supplementary material for: Implementing Blockchains for Efficient Health Care: Systematic Review
Source: J Med Internet Res. 2019 Feb 12;21(2):e12439. doi: 10.2196/12439 (PMC6390185; doi:10.2196/12439)
Supplement: Multimedia Appendix 6 [file jmir_v21i2e12439_app6.docx]

Multimedia Appendix 6

| **Reason for exclusion** | **Number of articles excluded** |
| --- | --- |
| Duplicates | 6 |
| Inaccessible | 7 |
| Too focussed on financial aspects | 7 |
