# Supplementary material for: Implementing Blockchains for Efficient Health Care: Systematic Review
Source: J Med Internet Res. 2019 Feb 12;21(2):e12439. doi: 10.2196/12439 (PMC6390185; doi:10.2196/12439)
Supplement: Multimedia Appendix 7 [file jmir_v21i2e12439_app7.docx]

Multimedia Appendix 7

| **Company / System Name** |
| --- |
| MedRec |
| Factom |
| Medicalchain |
| Medvault |
| phrOS |
| Guardtime |
| PokitDok / DokChain |
| GemHealth / GemOS |
| Patientory |
| Blockchain Health Co |
| Healthcombix |
| IBM Watson (Health) |
| BurstHQ |
| YouBase |
| HealthNautica |
| Philips Blockchain Lab |
| Hashed Health |
| Humana, Optum, United Healthcare, Multiplan, Quest Diagnostics (collaboration) |
| SimplyVital |
| Medable (Insight) |
